# Harvesting Microalgae Biomass Using Magnetic Nanoparticles from Iron-Rich Particulate Material

**DOI:** 10.1021/acsomega.5c03941

**Published:** 2026-02-09

**Authors:** Ana Carolina de Lima Barizão, Larissa Lamburghini Brandão, Giovanna Pinto Pires, Luiz Eduardo de Oliveira Gomes, Jairo Pinto de Oliveira, Sérvio Túlio Cassini

**Affiliations:** † Department of Environmental Engineering, 28126 Federal University of Espírito Santo, Fernando Ferrari avenue, 514, Vitória, Espírito Santo Cep: 29075-910, Brazil; ‡ Department of Industrial Chemistry, Federal Institute of Espírito Santo, Min. Salgado Filho avenue, 1000 Vila Velha, Espírito Santo Cep: 29106-010, Brazil; § Department of Biotechnology (Renorbio-UFES), Federal Institute of Espírito Santo, Maruípe avenue Vitória, Espírito Santo Cep: 29053-360, Brazil; ∥ Department of Morphology, Federal University of Espírito Santo, Maruípe Avenue Vitória, Espírito Santo Cep: 29053-360, Brazil; ⊥ Laboratory of Physical Chemical and Microbiological Characterization, Research Innovation and Development Center, Eliezer Batista Avenue Cariacica, Espírito Santo Cep: 29140-500, Brazil; # Postgraduate Program in Environmental Oceanography (PPGOAM), Department of Oceanography and Ecology, Federal University of Espírito Santo, Vitória, Espírito Santo Cep: 29075-910, Brazil; ¶ Laboratory of Environmental Geochemistry and Marine Pollution, Department of Oceanography and Ecology, Federal University of Espírito Santo, Fernando Ferrari Avenue, 514 Vitória, Espírito Santo Cep: 29075-910, Brazil

## Abstract

Using magnetic nanoparticles for efficient microalgae harvesting has shown promising results with enhanced harvesting efficiency within a reduced time frame. However, the cost of nanoparticle synthesis presents a potential constraint the widespread implementation of this technique. In this way, this work investigated, for the first time, the efficacy of magnetic nanoparticles (MNPs) derived from particulate matter for harvesting microalgae (*Chlorella* sp.) both naked and functionalized with commercial tannin. The nanoparticles were characterized using X-ray diffraction (XRD), transmission electron microscopy (TEM), scanning electron microscopy (SEM), energy dispersive X-ray spectroscopy (EDS), Fourier transform infrared (FTIR), and zeta potential. The MNPs present in the particulate material were magnetite with an estimated size (based on 1000 particles) of around 30.5 ± 9.56 nm. Harvesting efficiency (HE %) was optimized using full factorial experiments, in which the most influential variables (pH and MNPs concentration) were combined at different levels, achieving optimal harvesting efficiency. The MNPs (naked) exhibited higher harvesting efficiency (HE % = 86%; MNPs concentration = 1250 mg. L^–1^; pH = 3) than functionalized nanoparticles (HE % = 77%; MNP-TANs concentration = 1100 mg/L; pH = 3.5); however, a higher MNPs concentration was necessary in a lower pH. The functionalization contributed to particle stabilization, increasing its reuse cycles from 3 (MNSs) to 7 cycles (MNP-TANs). At pH 10 (the final pH of the microalgae cultivation), both exhibited a similar HE % of 60%.

## Graphical abstract

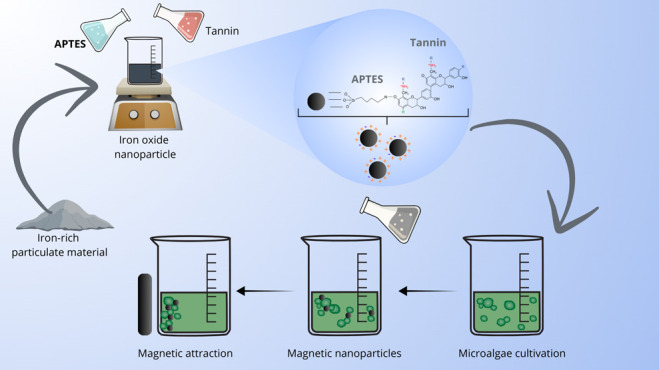

## 1 Introduction

The increasing frequency and intensity of extreme climate events underscore the urgent need to transition from fossil fuels to renewable energy sources as a key strategy to mitigate ongoing climate change scenarios.^1^ In this context, microalgae biomass production has emerged as a promising renewable and sustainable energy source for biofuel production. The potential for large-scale biomass results from microalgae’s high CO_2_ fixation rates during photosynthesis and their high growth rates. Furthermore, microalgae can be produced without arable land.^1^ Other uses of microalgae include nutrient and contaminant removal (e.g., microalgae production in wastewater treatment plants) and the potential generation of carbon credits to achieve regional to international climate agreements.^2^

A key issue for the large-scale production of microalgae is performing the harvesting step efficiently, quickly, and cheaply. The morphological characteristics of microalgae, such as a high negative surface charge and low density make it difficult to reach the optimum biomass concentration stage.^3^ To overcome this limitation, methods like filtration,^4^ flocculation,^5^ and centrifugation^6^ have been developed over the years. In this scenario, the use of reusable magnetic nanoparticles (MNPs), e.g., magnetite (Fe_3_O_4_), has been highlighted to provide fast and efficient harvesting, resulting in high-quality biomass without residual contamination from the harvesting stage.^7^

Brazil has one of the largest mineral reserves globally and is the second country with the largest iron reserves globally (19.6% of the global reserves or 33.000 tons of iron). The extraction of metallic substances represents 89% of the mineral commercialized production, where iron ore accounts for 80.1% of this total. Pará and Minas Gerais are Brazil’s main gross producers of iron, while the state of Espírito Santo is the leader in iron ore processing, beneficiation, and selling to the international market.^8^

During the transport, processing, and storage of iron, small particles can be released into the air and become part of the particulate material composition.^9^ The appropriate application of this nanomaterial can be viwed through a circular economy lens, providing an economically efficient destination while reducing costs associated with microalgae harvesting and improving human health. In addition, functionalization can be used to modify the nanoparticle’s surface and improve their properties, favoring the interaction between magnetic nanoparticles MNPs and microalgae.^10,11^

Some commercial flocculants derived from tannins extracted from Acacia trees are already used in water treatment. In this case, tannins are chemically modified by inserting amino groups into their structure, which makes them more positive.^12^ If used as an MNP functionalizer, tannin can interact with microalgae cells, forming magnetic flocs quickly harvested by an external magnetic field.^13^ This study aimed to evaluate, for the first time, the potential of magnetic nanoparticles (MNPs) derived from iron-rich particulate matter for harvesting microalgae (*Chlorella* sp.). The study compared their performance in both bare form and after functionalization with commercial tannin, aiming to improve stability, reusability, and harvesting efficiency.

## 2 Materials and Methods

### 2.1 Materials

Particulate material collected at Guarapari, ES, Brazil (20°39′14.48′′ S; 40°29′14.85′′ W), 3-(Aminopropil)­trietoxisilano (APTES), commercial tannins, Ultrapure Water (Sartorius), Neodymium magnet 50 × 50 × 12 mm (Supermagnet, Brazil). *Chlorella* sp. strain (L06, from Laboratory of Chemical, Physical, and Microbiology Characterization of Federal University of Espirito Santo). BG11 medium (NaNO_3_ (1.5 g L^–1^); K_2_HPO_4_ 3H_2_O (40 mg L^–1^); MgSO_4_ 7H_2_O (75 mg L^–1^); CaCl 2H_2_O (36 mg L^–1^); C_6_H_8_O_7_ (6 mg L^–1^); C_6_H_5_FeO_7_ (6 mg L^–1^); Na_2_CO_3_ (20 mg L^–1^); (Andersen, 2005). Laboratory glassware includes beakers, volumetric flasks, and Falcon tubes. All glassware was sanitized using aqua regia (HCl: HNO_3_) 5 times and washed ten times with ultrapure water before the experiments.

### 2.2 Selection of Magnetic Nanoparticles Source and Functionalization

Nanoparticle sources were selected based on the literature, considering the availability, costs, and magnetic iron oxide content. For 15 days, the particulate matter (selected source) was collected on a clean surface (open area (15 m^2^)) in Guarapari, Espírito Santo, Brazil. The raw material was first screened to remove associated materials, such as leaves and dust. The remaining particles were resuspended in 200 mL of ultrapure water and left to stir for 1 min. The resulting solution was subjected to a magnetic field influence for 1 min separating the magnetic nanoparticles. The washed nanoparticles were left in a muffle (500 °C) for 30 min to eliminate the remaining organic content.

For stabilization, 0.6947 g of MNPs were dispersed in 25 mL of ethanol containing 3 mL of APTES and stirred for 10 min (room temperature). In sequence, 0.6947 g of MNPs were incorporated into the mixture, and continuous stirring was performed for 48 h. The resulting material was washed several times until all unreacted APTES was entirely removed from the supernatant. The functionalization step using commercial tannins was based on the method described by Zhao et al.^14^ In this stage, 1 g of MNPs was combined with 40 mL of a tannin solution (25 g L^–1^) and the mixture was stirred at 25 °C for 2 h. The tannin-functionalized nanoparticles (MNP-TANs) were then washed repeatedly until the supernatant was free of excess tannins and subsequently stored in Falcon tubes at 8 °C and protected from light.

### 2.3 Characterization of Magnetic Nanoparticles

The magnetic nanoparticles were characterized by using a combination of analytical techniques. The crystalline phases were determined by X-ray diffraction (XDR) (XRD-6000, Shimadzu) within the 2θ range 20–70°, using a voltage of 40 kV and a step size of 0.02°. Morphology, size, dispersion, and elemental composition were examined by transmission electron microscopy (TEM, JEOL JEM-1400; 100 kV, 3 mm working distance, 20 μA current) and scanning electron microscopy (SEM, JEOL JSM-6610LV; 15 kV, 30 μA), equipped with an energy-dispersive X-ray (EDS) detector. Functionalization was evaluated by Fourier-transform infrared spectroscopy using an Agilent Cary 630 FTIR equipped with a diamond ATR crystal (32 scans, 2 cm^–1^ resolution). An automatic baseline correction was applied to eliminate spectral drift and background noise, enabling accurate identification of the characteristic vibrational bands. CHNS elemental analysis (Thermo Scientific FlashSmart 2000) was carried out using sulphanilamide as the calibration standard (N, 16.27%; C, 41.84%; H, 4.68%; O, 18.58%; S, 18.62%), with measurements performed in triplicate. Additionally, the surface charge of both the nanoparticles and *Chlorella* sp. cells was determined by zeta potential analysis using a Litesizer 500, at pH 4, 7, and 10, with 50 measurement runs, in aqueous suspension (approximately 25 °C).

### 2.4 Molecular Docking

The docking analysis evaluated the nanoparticle interaction mechanisms with their stabilizer (APTES) and functionalizer (tannin). First, the magnetite supercell (2 × 2 × 2) and the tannin molecule were both modeled in Avogadro,^15^ with the latter based on the two-dimensional polymeric structure.^16^ The magnetite supercell was subjected to the UFF force field, while tannin was separately treated with four different force fields (GAFF, Ghemical, MMFF94, and UFF). Overall, five distinct PDB files were obtained, although this work focuses exclusively on the docking experiments of MNPs with commercial tannin under the Ghemical force field, as it has shown the best results in another work being developed by the team.

The magnetite supercell was prepared with AutoDock Tools 1.5.7 as a receptor for docking experiments. The preparation steps included removing water,^17^ adding polar hydrogen atoms^17–19^ calculating Gasteiger charges,^20^ and assigning the macromolecule’s atoms the AD4 atom type. Meanwhile, tannin was prepared as the ligand. Since it presented over 100 torsions, surpassing the allowed maximum of 32, the number of torsions set was 32. Both molecules were saved as PDBQT files.

Since AutoDock Vina 1.2.3 does not support PDBQT files that contain HETATM and CONECT records, docking was performed by AutoDock 4.2, with 7.964, 9.091, and 10.017 as the *x*, *y*, and *z* centers, respectively; 90 as box size for all three coordinates; grid spacing of 0.375 Å; and 100 runs, following the protocol of Rizvi et al. (2013).^19^ The binding energies and RMSD values were extracted from the result tables in the DLG file, and an in-house R script extracted the intermolecular energy values from the runs. The energy and RMSD plots were generated using the ggplot2 package^21^ in R 4.2.2, within the RStudio/Posit 2023.03.1 + 446 environment. Three-dimensional images illustrating the interactions between the magnetite supercell (representing the iron­(III) oxide nanoparticle capped with APTES) and commercial tannins were rendered using AutoDock Tools, ChimeraX, and PyMOL.

### 2.5 Factorial Experiments Design

*Chlorella* sp. was cultivated in batch mode using 5 L tubular photobioreactors filled with BG11 medium NaNO_3_ (1.5 g L^–1^); K_2_HPO_4_ 3H_2_O (40 mg L^–1^); MgSO_4_ 7H_2_O (75 mg L^–1^); CaCl 2H_2_O (36 mg L^–1^); C_6_H_8_O_7_ (6 mg L^–1^); C_6_H_5_FeO_7_ (6 mg L^–1^); Na_2_CO_3_ (20 mg L^–1^; Andersen, 2005), under constant stirring at 70 rpm and natural illumination of approximately 1500 l×. Microalgal growth was monitored at 680 nm until the cultures reached the stationary phase. The biomass obtained at this stage was subsequently used in the factorial experiments.

To optimize microalgae harvesting, a full factorial (2^3^) was realized. The independent variables pH, nanoparticle concentration, and their levels (Table 1) were selected based on an extensive literature review, resulting in the design matrix in Table 2. The nanoparticles were tested in their naked version (MNPs) and functionalized (MNP-TANs).

**Table 1:** Levels of Full Factorial Experiments

variables	levels		
	(−1)	0	(+1)
pH	4	7	10
MNPs concentration (mg. L^–1^)	800	1000	1200

**Table 2:** Design Matrix of Full Factorial (2^3^)

pH	naked MNPs/MNP-TAN concentration	treatments
–1	–1	2
–1	1	7
1	–1	11
1	1	3
–1	0	8
1	0	9
0	–1	4
0	1	10
0	0	5
0	0	6
0	0	13
0	0	12
0	0	1

All experiments were conducted in batch mode using 1 mL of microalgae suspension per assay. The harvesting efficiency (HE %, eq 1) was adopted as the dependent variable based on microalgae concentration in the supernatant after 1 min of reaction time (MNPs/microalgae) and 3 min of exposition to the magnetic field (680 nm). The adsorption capacity was already estimated under optimum conditions (*q*_exp_ (eq 2)).

1 $$HE\%=\left(C_{0}-C_{e}\right).\left(\frac{1}{C_{0}}\right).100$$

2 $$q_{\exp}=\left(\frac{C_{0}-C_{e}}{m}\right).V$$

where *C*_0_ and *C*_e_ correspond to the initial and final dye concentrations (mg L^–1^), *V* is the solution volume (L), and *m* is the mass of the adsorbent (g).

### 2.6 Isotherms and Thermodynamic Parameters

The adsorption isotherms were obtained under the previously established optimal conditions (MNPs concentration = 1250 mg L^–1^; pH = 3/MNP-TANs concentration = 1100 mg/L; pH = 3.5) with different microalgae concentrations ranging from 200 mg L^–1^ to 3000 mg L^–1^, and temperature at 25, 35, and 45 °C. After the reaction time, the solutions were exposed to a magnetic field for 3 min, and the HE % was estimated based on microalgae concentration in the supernatant (680 nm). The Langmuir (eq 3) and Freundlich (eq 4) models were fitted to the experimental data to determine which provided the best correlation.

3 $$q=\frac{q_{\max}K_{L}C_{e}}{1+}K_{L}C_{e}$$

4 $$q=K_{F}{C_{e}}^{1/n}$$

where *q* is the solute adsorbed per gram of adsorbent at equilibrium (mg g^–1^), *q*_max_ is the maximum adsorption capacity (mg g^–1^), *K*_L_ is the interaction constants between adsorbate and adsorbent (mg L^–1^) and *K*_F_ Freundlich adsorption capacity constant (mg L^–1^), *C*_e_ is the concentration of adsorbate at equilibrium (mg L^–1^), and 1/*n* the surface heterogeneity constant.^22^

The thermodynamic parameters were also determined at temperatures of 25, 35, and 45 °C. Eqs 5–7 determined the Gibbs free energy variation (Δ*G*°), enthalpy (Δ*H*°), and entropy (Δ*S*°).

5 $$\Delta G^{{}^{\circ}}=-RT\ln K$$

6 $$\Delta G^{{}^{\circ}}=\Delta H{\;}^{{}^{\circ}}-T{\Delta S}^{{}^{\circ}}$$

7 $$\ln K=\frac{\Delta S^{{}^{\circ}}}{R}-\frac{\Delta H^{{}^{\circ}}}{R^{x}}\frac{1}{T}$$

where *R* is the ideal gas constant (8.3144 J K^–1^ mol^–1^), *T* is the temperature in Kelvin, and *K* is the equilibrium constant.^23^

### 2.7 Reuse of Nanoparticles

The reuse of MNPs and MNP-TAN was based on Lee et al. (2014).^24^ The reuse cycles were realized in the optimal conditions (MNPs concentration = 1250 mg L^–1^; pH = 3/MNP-TANs concentration = 1100 mg/L; pH = 3.5). After the reaction time (1 min), the solutions were exposed to an external magnetic field for 3 min. HE % was estimated based on microalgae concentration in the supernatant (680 nm). The flocks (nanoparticles/microalgae) obtained in any harvesting cycle were suspended in 300 μL of distilled water at pH 12 and vortexed for 30 s, followed by 1 min in ultrasound. The recovered nanoparticles were separated by a magnetic field, washed, and recycled during ten cycles.

## 3 Results and Discussion

### 3.1 Selection of MNPs Source and Functionalization

First, a literature review was conducted to identify possible nanomagnetic iron sources, considering the iron content, positive and negative points, and the costs of material requirements, if available (Supporting Information, Table S1). Based on these analyses, the particulate material was selected due to its easy acquisition, simple pretreatments, local availability, and, different from others, that do not have negative points capable of limiting their potential application in microalgae harvesting, for example, heavy metals that can contaminate the biomass.

The material was collected in the metropolitan area on the southeast coast of Brazil, in Espírito Santo. The region is highly industrialized, including steel and iron pelletizing companies, which are responsible for approximately 70% of all particulate material emissions in the region.^9^ Galvão et al. (2022) reported the presence of iron oxide in the particulate matter from this region, with magnetite accounting for up to 4% of the settable particulate matter, reaffirming the potential of this source for magnetic nanoparticle acquisition. Furthermore, we are unaware of any other studies that have recognized particulate matter as a potential source of magnetic nanoparticles for harvesting microalgae.^9^

#### 3.1.1 Characterization of Magnetic Nanoparticles

The collected particulate material, after being washed, showed a yield of 23%, and after muffle furnace treatment, the yield decreased to 18%. This reduction is attributed to the high number of impurities and dust particles that were deposited along with the material during the collection process. Through elementary analysis, it was observed that the muffle process was able to reduce C, passing from 6.135% to 1.956%. The N (0.472%) and H (0.816%) were also reduced to 0.277% and 0.363%, respectively. Despite efficiency, the process did not completely remove impurities, which can make the nanoparticles more heterogeneous.^25^

The magnetic fraction of the particulate material (MNPs) was analyzed by TEM (Figure 1A). The particles presented an estimated size (based on 1000 particles) around 30.5 ± 9.56 nm (Figure 1B), with an aspect ratio (AR = 1.12 ± 0.5) indicating quasi-spherical (Figure 1C). However, the hydrodynamic size (Figure 1D) observed was 295 ± 40 nm, which could be related to the agglomerate tendency of the material in aqueous solution or the polydispersity (Pdl = 1.0). The polydispersity of MNPs was expected since nanoparticles were obtained from natural sources and did not undergo any size selection process.^26^ Elevated polydispersity often promotes aggregation and even sedimentation, which reduces the accessible surface area and, consequently, the adsorption capacity.^27^

**Figure 1:**
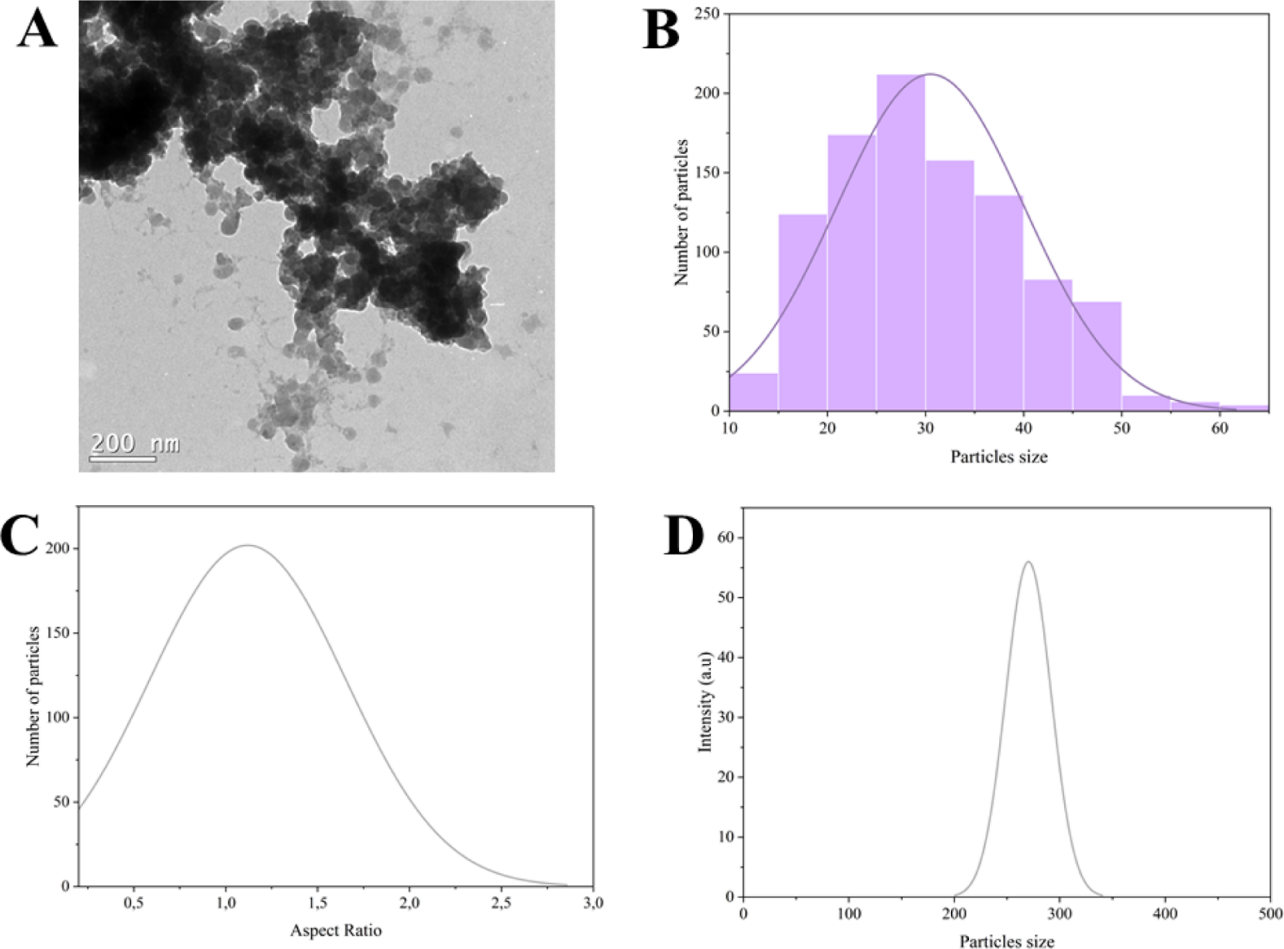
(A) TEM of magnetic fraction of particulate material; (B) histogram of MNPs size distribution, based on Figure 1A; (C) aspect ratio of MNPs, based on Figure 1A; (D) hydrodynamic size of MNPs.

Comparing the XDR diffractogram of MNPs from particulate material with the synthetic magnetite (Fe_3_O_4_) diffractogram, it is possible to observe similarities, with peaks in 35,46°, 56,6°, and 62,60°, corresponding to (311), (422), and (440)^28^ (Figure 2A). The additional peaks observed may be attributed to residual impurities remaining after the washing and muffle treatment. The SEM images confirmed the size of the synthesized nanoparticles (red square). However, due to the strong aggregation tendency of the MNPs, pronounced agglomeration can be observed. Agglomeration is one of the most common phenomena reported for iron oxide nanoparticles, primarily driven by attractive van der Waals forces and anisotropic magnetic dipole–dipole interactions.^29^ Elemental analysis of the MNPs performed by SEM coupled with EDS further supported this behavior (Figure 2B,C). In addition to the high Fe and O contents, signals corresponding to silicon, sodium, gold, calcium, magnesium, and aluminum were detected. The presence of gold is due to the sample preparation procedure.

**Figure 2:**
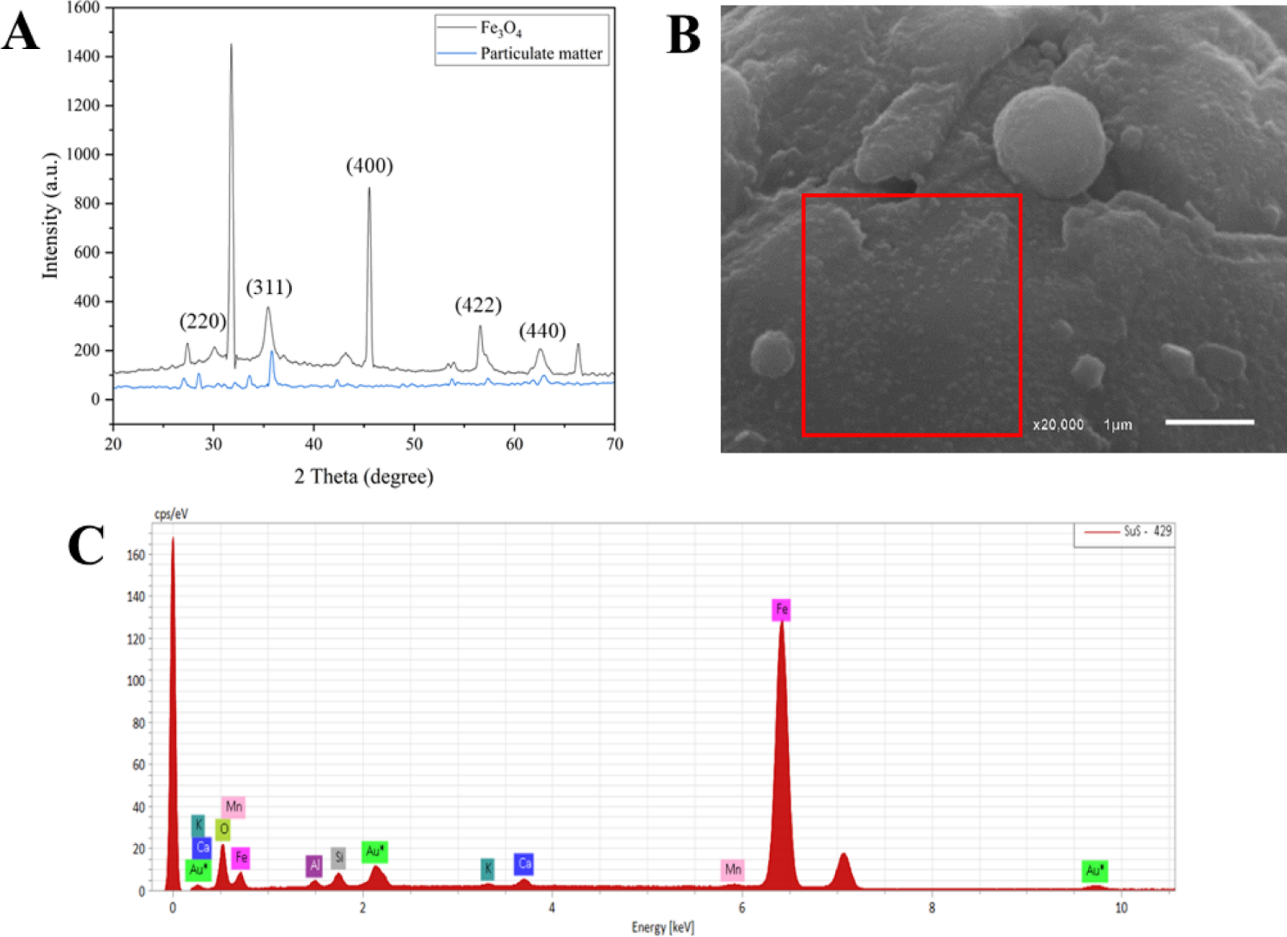
(A) XDR diffractogram of MNPs; (B) SEM of MNPs; (C) EDS of MNPs.

The obtained MNPs were stabilized by APTES and functionalized by using commercial tannins (MNP-TANs). The elementary analysis (Table 3) evidenced an increase in the N content of functionalized samples. This occurred due to the presence of N in APTES and to the amines in the tannins’ structure, which confer a positive charge.

**Table 3:** Elementary Analyses of MNPs and MNP-TANs

samples	nitrogen	carbon	hydrogen
MNPs	0.277	1.956	0.363
MNP-TANs	0.312	2.071	0.463
commercial tannin	5.855	35.821	6.420

In addition, the bands 1306 and 1289 cm^–1^ observed in the FTIR (Figure 3) can be related to aromatic C–N stretching or NO_2_ symmetric stretching, indicating the presence of tannins. These nitrogen-containing groups likely arise from chemical modifications via amine-based reactions, which are known to enhance the flocculation performance of tannins. At the same time, the bands 1800 and 1770 may be due to C=O stretching, supporting the above supposition. A bands at 3024 cm^–1^ and 2982 cm^–1^, fall within the typical region of aromatic C–H stretching vibrations. These features are consistent with the organic moieties (tannin) introduced onto the nanoparticle surface.^30^

**Figure 3:**
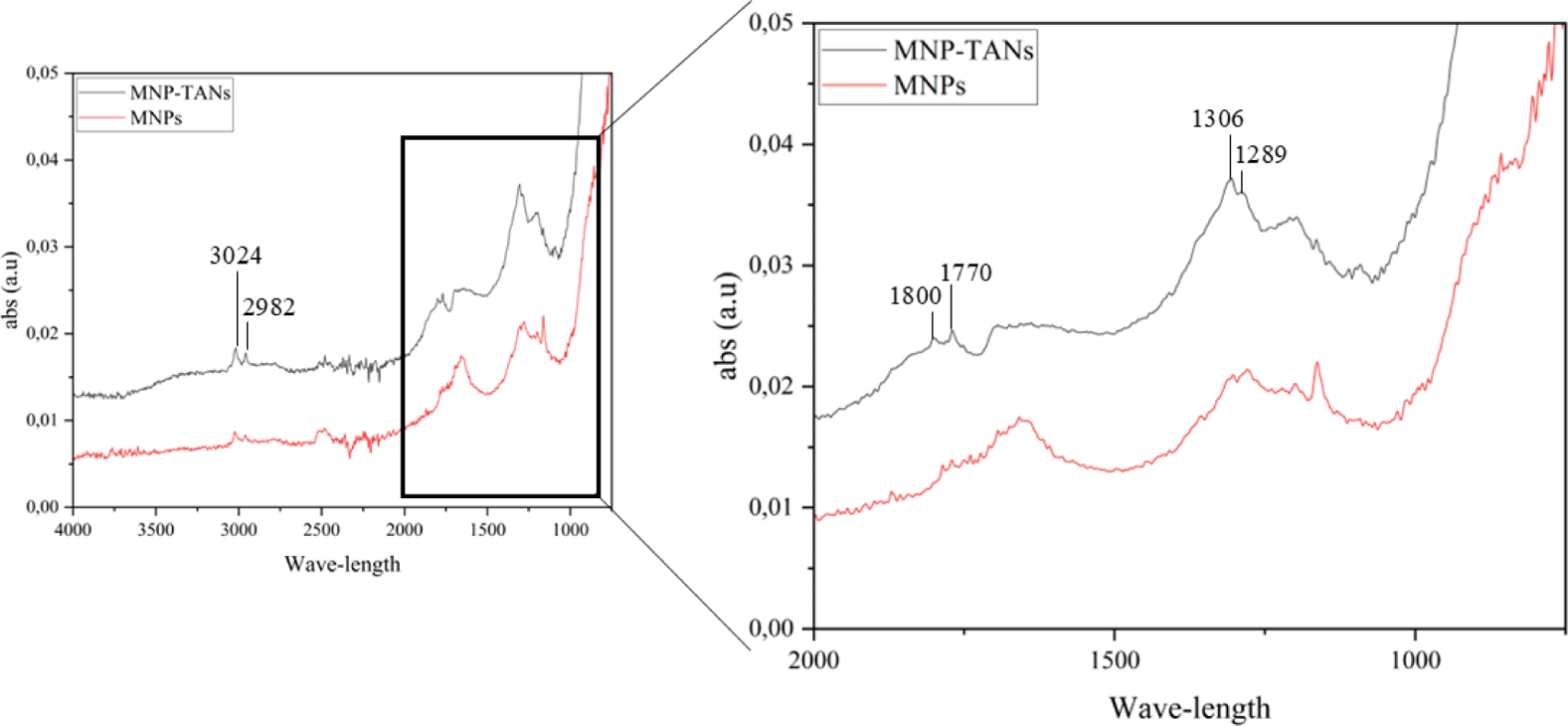
FTIR of MNPs and MNP-TANs.

### 3.2 Molecular Docking

This type of molecular modeling technique demonstrates how molecular structures fit together; a useful tool in the comprehension of the protein’s behavior.^31^ However, in this work, this effective tool was applied to understand the interaction between the magnetic nucleus of nanoparticles (Fe_3_O_4_) and the stabilizer/functionalizer molecules (MNP-APTES-tannins). The binding and intermolecular energy values of the hundred runs are shown in Figure 4, below.

**Figure 4:**
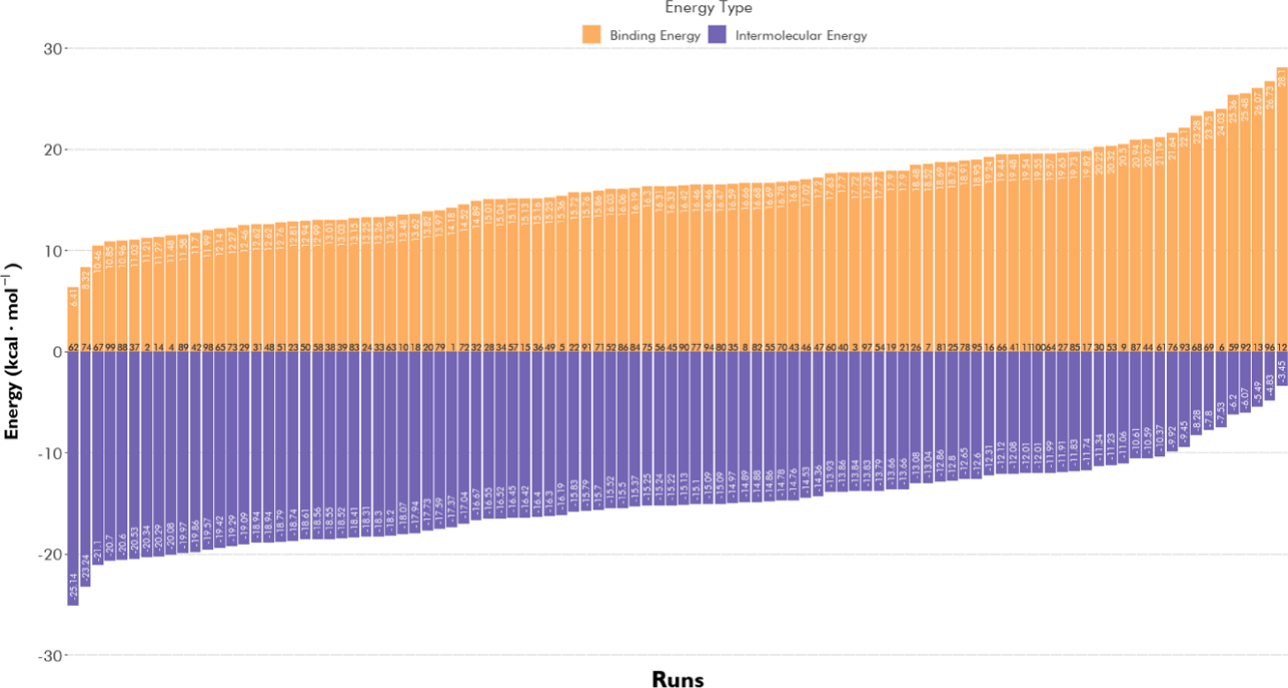
Main energies involved in the interaction between commercial and magnetite (stabilized with APTES). The orange bars refer to the binding energy and the purple bars to the energy of intermolecular interactions, whose final value is a sum of the energies of hydrogen bonds, Van Der Waals, solvation, and electrostatics in AutoDock 4.2. Each bar corresponds to one of the hundred runs realized. Energy types involved in MNP-APTES-TAN bonds.

Commercial tannin presented a high root-mean-square deviation (RMSD; it measures how much the position of the ligand atoms deviated from the position of the first mode).^32^ Varying between 35 and 55 Å, in a hundred runs (Figure 5). This could be related mainly to the use of a modeled molecule (and not crystallographic), making the result vary according to the defined configurations. The green points represent the worst (point 12) and best (point 62) results for the energy of intermolecular interactions.

**Figure 5:**
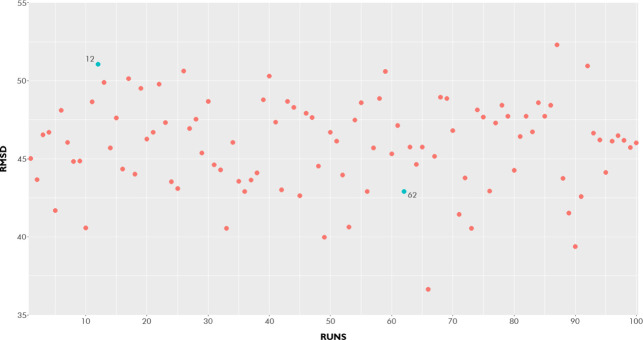
Root mean square deviation (Å) of each one of the hundred conformations of commercial tannin (ligand).

The intermolecular energies were predominant (more negative, indicating a spontaneous process). In Figure 6A,B, it was observed that hydrogen bonds may be occurring between the O from magnetic iron oxide and the oxo from tannin. The dipole–dipole can also be considered in this case since Fe_3_O_4_ is a polar nanoparticle (due to differences in charges caused by the distribution of Fe^2+^ and Fe^3+^, as well as tannin, which is composed of the bond of flavan-3-ol).^33,34^ Thus, both can present negative and positive regions, leading to their interactions. It is also important to stress that the presence of impurities in the MNPs, even after pretreatment, may have interfered with this interaction in ways that we could not predict. The prevalence of weaker bonds does not necessarily indicate the inefficiency of the functionalizing layer or that it will easily detach after the use of nanoparticles..

**Figure 6:**
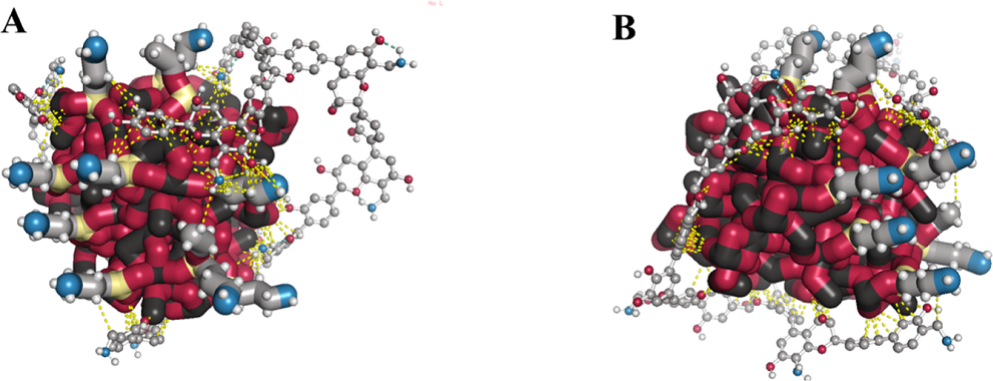
MNP-TAN molecular docking (MNP-APTES-TAN bonds); being atoms in red (O), black (Fe), gray (C), blue (N), white (H), yellow (Si), and dashed yellow line (intermolecular interactions). (A) Original molecule position; (B) rotated molecule (45° in *Y* axis).

#### 3.2.1 Factorial Experiments Design

The Full factorial experiments were carried out to optimize the harvesting of *Chlorella* sp. The biomass harvested was cultivated for 8 days (the end of the exponential phase), achieving a biomass concentration of 1.5 g. L-1 (pH = 10.7). According to the Pareto chart, for both nanoparticles (MNPs and MNP-TANs), the two variables were significant (Figure 7A–C, and C; ANOVA results in Supporting Information, Tables S2 and S3); however, the pH was the most influential in all cases.

**Figure 7:**
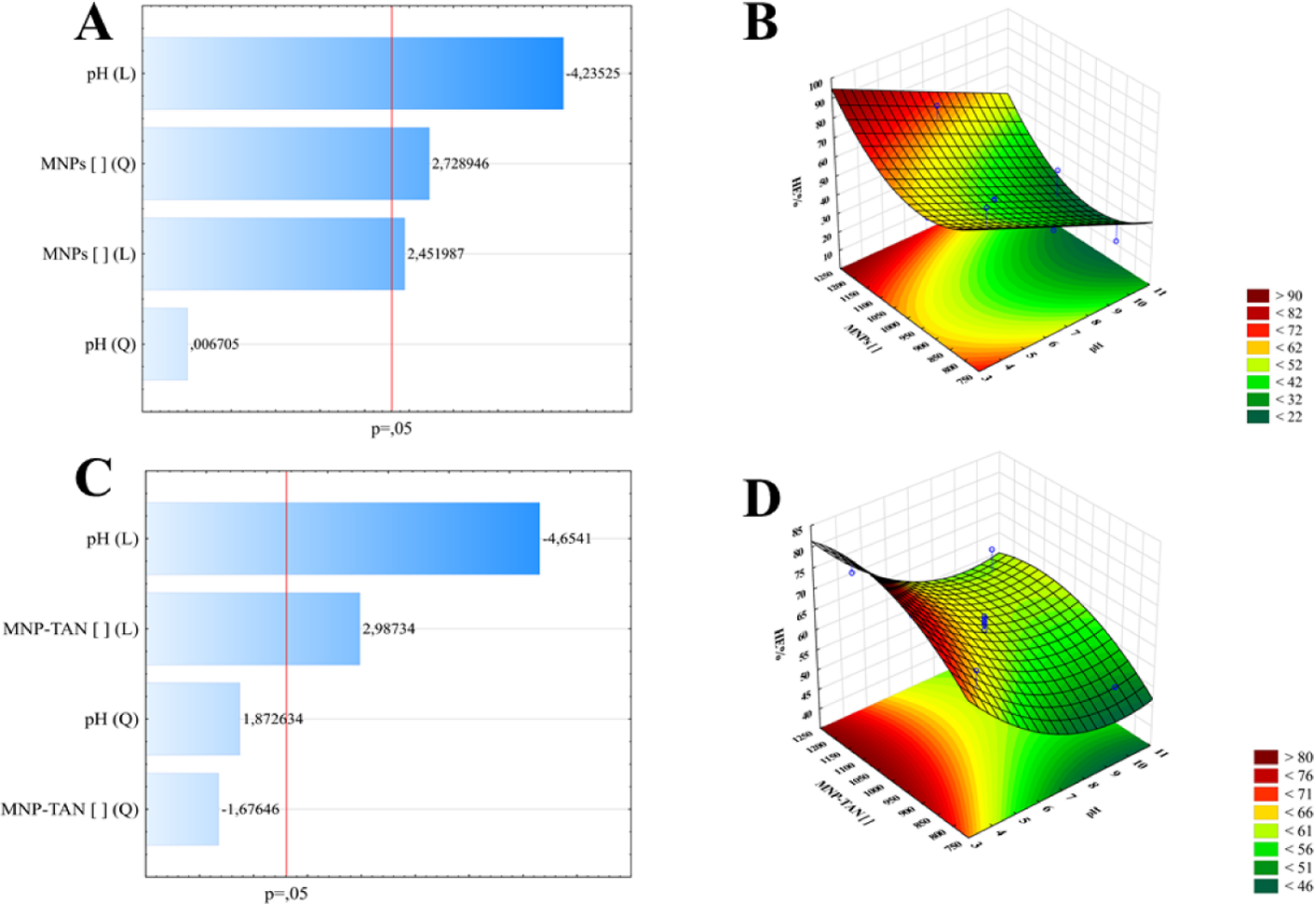
(A) Pareto chart from MNPs; (B) response surface chart from MNPs; (C) pareto chart from MNP-TANs; (D) response surface chart from MNP-TANs.

The model demonstrated an acceptable adjustment to the data, with *R* ^2^ = 0.80 (MNPs) and *R* ^2^ = 0.81 (MNP-TANs) (Figure 7A,B). The MNPs presented higher HE % (93.5%) than MNP-TANs (82%), however, the nanoparticles concentration necessary was higher (1250 mg L^–1^), and the pH (3) was smaller compared to MNP-TANs (1100 mg L^–1^ at pH = 3.5), achieving a near adsorption capacity being *q*_exp_ = 1.12 g mg^–1^ for MNPs and *q*_exp_ = 1.025 g mg^–1^ for MNP-TANs. The experimental HE % obtained in optimal conditions was slightly lower than that from the model, being HE % 86% for MNPs and HE % = 77%. This can be justified by the value of *R* ^2^, below 90 in both cases.

The higher HE % of MNP-TANs can be justified by their less negative charge (−4 mV) compared with MNPs (−10 mV) (Figure 8). The presence of amine groups on the MNP-TANs surface may have favored attraction with microalgae, requiring a lower concentration of nanoparticles. It is also essential to consider that naked nanoparticles may return to their iconic form due to oxidation by low pH, which does not occur in MNP-TANs due to their double protective layer (APTES-tannin). Both HE % tended to decrease when the pH was increased to 10, which makes sense as both become more negative, increasing the repulsion with microalgae. However, even though they become slightly negative at pH 10, they could maintain their HE % ≈ 60%, a great result, considering that the final pH of microalgae cultivation is close to 10.

**Figure 8:**
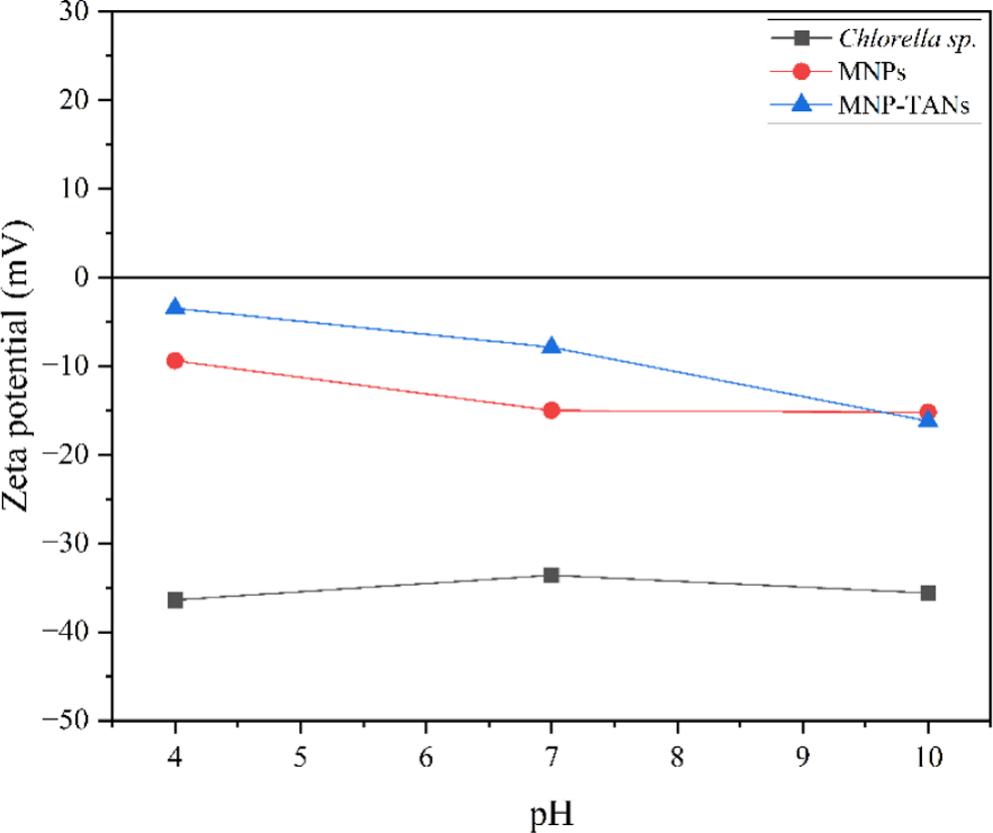
Zeta potential from MNPs, MNP-TANs, and *Chlorella* sp.

#### 3.2.2 Isotherms and Thermodynamic Parameters

The adsorption isotherm models are essential to describe the interaction between the adsorbent and adsorbate at equilibrium and constant temperature (Saleh, 2022). For MNPs, increasing temperature negatively influences the HE %, where the best temperature is 25 °C, indicating an exothermic process (Figure 9A). Freundlich was the best-fitting model (Figure 9B) with a great adjustment to the data (*R* ^2^ = 0.98). The Freundlich model considers a heterogeneous solid, where some adsorption sites are highly energetic and the adsorbent/adsorbate interaction is strong. In contrast, others present low energy, consequently weakening the bond.^13^

**Figure 9:**
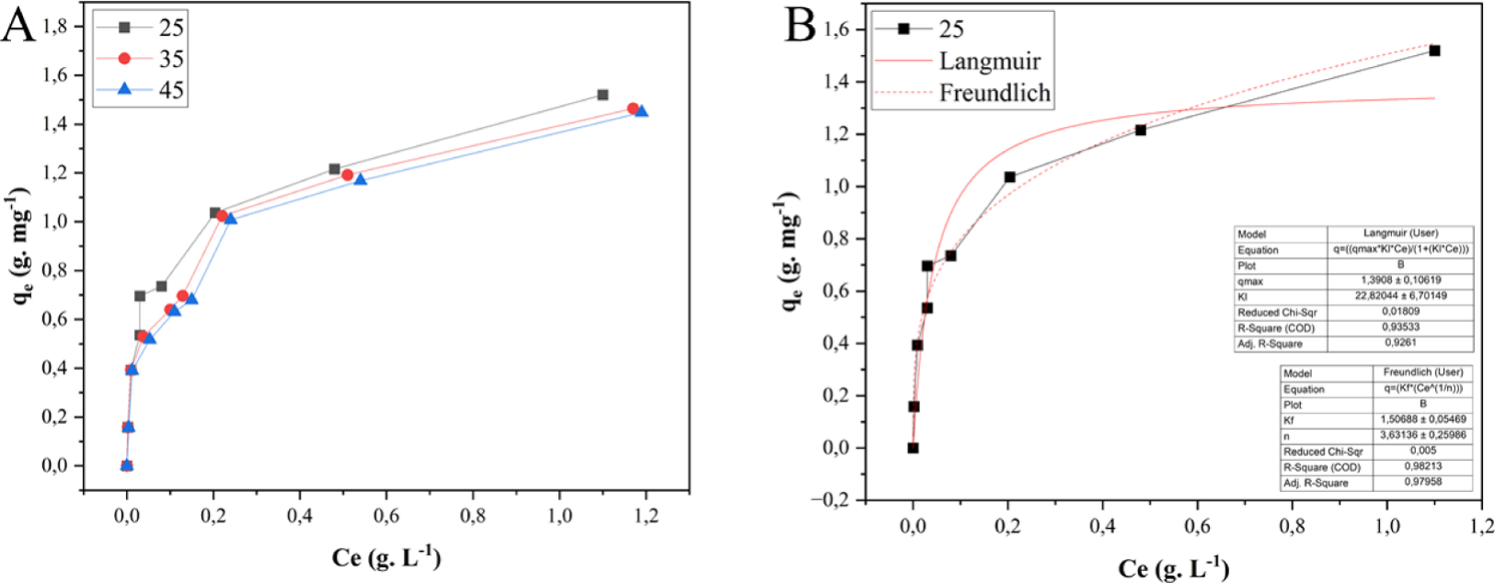
(A) Adsorption isotherms (MNPs) at 25 °C, 35 °C, and 45 °C; (B) adsorption isotherm (MNPs) at 25 °C and isotherm models of Langmuir and Freundlich.

These results make sense considering that MNPs are naked nanoparticles from natural sources and contain impurities (molecules other than Fe and O) in their structure, as observed in the characterization, which can interfere with the adsorption process. Despite this, it was not enough to disable the adsorption since (*n* > 1; 3.63 ± 0.25) indicates a favorable process. The maximum adsorption capacity of the adsorbent (*q*_max_ = 1.5 g mg^–1^) was compatible with those obtained in factorial experiments (*q*_exp_ = 1.12 g mg^–1^).

Like MNPs, the adsorption process with MNP-TANs was also exothermic (Figure 10A). Exothermic adsorption can reduce industrial costs by decreasing the need for supplementary energy sources. The MNP-TANs, the best-fitting model with data, was the Langmuir (Figure 10B), with *R* ^2^ = 0.94. Different from Freundlich’s model, Langmuir’s model assumes adsorption in a monolayer, where the bonded sites have equivalent energy (allowing only one molecule at each site) (Saleh, 2022). In this case, the functionalization process may have contributed to a more uniform particle surface, resulting in active sites with closer binding energies, different than the naked particle. The maximum adsorption capacity of the adsorbent was (*q*_max_ = 1.33 g mg^–1^), also compatible with those obtained in factorial experiments (*q*_exp_ = 1.02 g mg^–1^).

**Figure 10:**
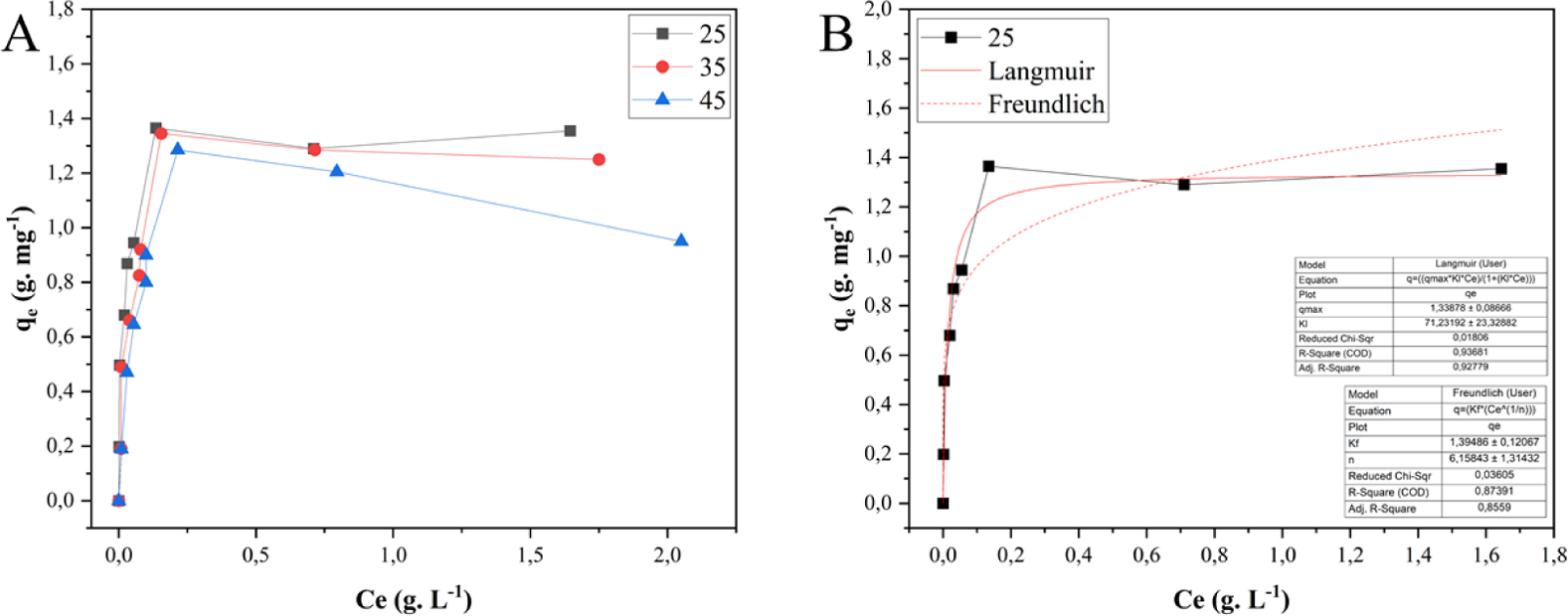
(A) Adsorption isotherms (MNP-TANs) at 25 °C, 35 °C, and 45 °C; (B) adsorption isotherm (MNP-TANs) at 25 °C and isotherm models of Langmuir and Freundlich.

For the thermodynamic parameters (Table 4), the Gibbs free energy was negative for both adsorbents at all temperatures. Positive enthalpy and negative entropy can confirm spontaneous and favorable processes, while negative entropy indicates a reduction in the system’s disorder.

**Table 4:** Thermodynamic Parameters of *Chlorella* sp. Harvesting Process by MNPs and MNP-TANs

nanoparticles	Δ*G*° (kJ. mol^–1^)	Δ*H*° (kJ. mol^–1^)	Δ*S*° (kJ. mol^–1^)
MNPs	25 °C = −2.13	4.05	–0.00626
	35 °C = −2.24		
	45 °C = −2.00		
MNP-TANs	25 °C = −0.93	9.73	–0.0291
	35 °C = −0.83		
	45 °C = −0.33		

Based on our results, we propose a mechanism of interaction between microalgae and both types of nanoparticles. *Chlorella* cells are negatively charged at the working pH due to deprotonated carboxyl groups and phosphate groups on the cells, which promotes attraction to positively charged surfaces.^35^ The MNPs expose surface Fe–OH sites that near their point of zero charge are partially protonated (Fe–OH_2_^+^), enabling electrostatic attachment and inner-sphere ligand exchange with cell-wall groups, consistent with predominantly monolayer capture.^35^ After nanoparticles are functionalized, the organic shell introduces heterogeneous binding sites and additional interaction modes.^22^ Protonated amines (+NH_3_^+^) promote charge neutralization and patch attraction, while polyphenolic domains enable multidentate hydrogen bonding. This surface heterogeneity favors multisite, which is better captured by the Freundlich models.^22^ Foris et al. reported similar results using amine-functionalized MgFe_2_O_4_ nanoparticles to harvest *Chlorella vulgaris* *.* Harvesting efficiency ranged from 94.9% to 99.2%, with stronger nanoparticle–microalgae interactions observed at lower pH levels, where protonation intensifies electrostatic interactions.^35^

### 3.3 Reuse of Nanoparticles

The reuse of both nanoparticles was analyzed under optimal conditions. Despite the MNPs showing a higher HE % = 86%, this was only maintained during 3 reuse cycles (Figure 11A), dropping to less than 60% after the seventh cycle. The sharp decrease in HE % of MNPs in the third reuse cycle may be related to the dissolution of naked particles by the action of acidic pH. Iron oxide nanoparticles are easily destabilized at low pH, returning to the ionic form, as mentioned before. The MNPs sustained an HE % = 77% during 7 cycles, ending the last cycle with HE % ≅ 61% (Figure 11B). Proper functionalization of nanoparticles can enhance the overall efficiency of the reuse process by increasing the adsorption capacity, reducing material loss, and minimizing the need for frequent particle regeneration^34^.

**Figure 11:**
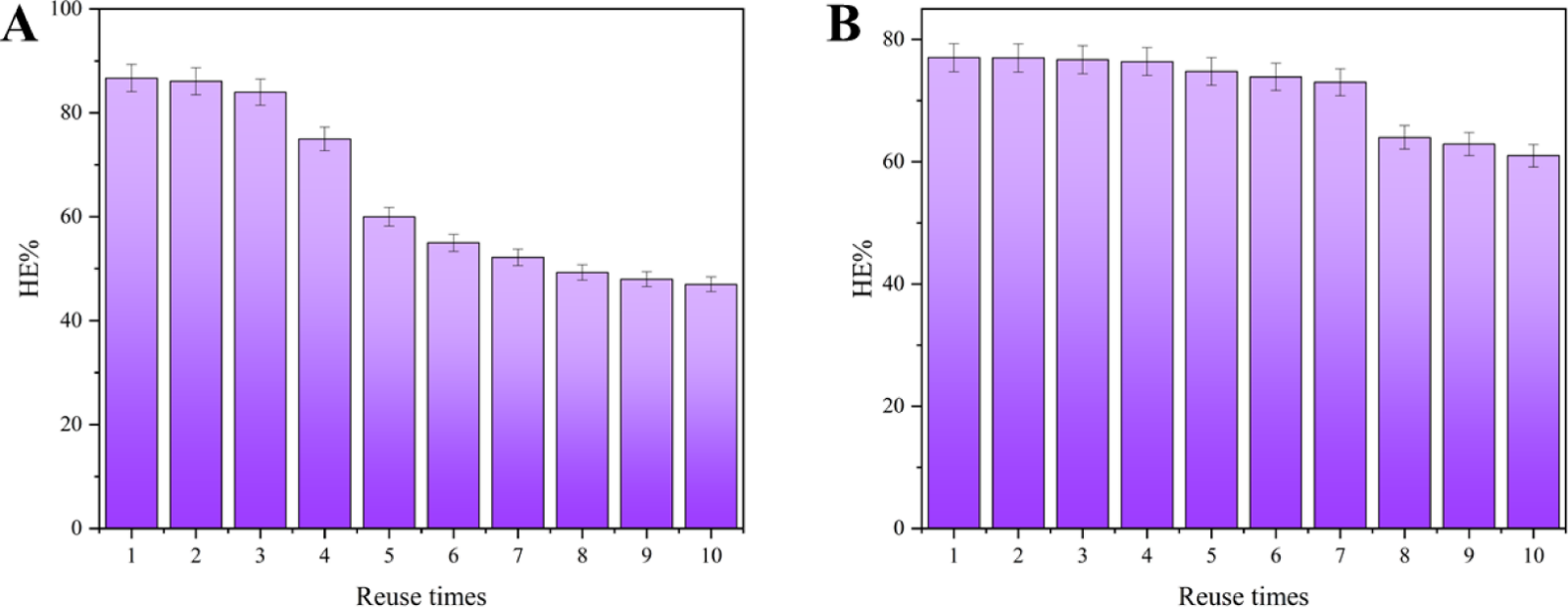
(A) Reuse cycles of MNPs applied in *Chlorella* sp. biomass harvesting; (B) reuse cycles of MNP-TAN applied in *Chlorella* sp. biomass harvesting.

## 4 Conclusions

Based on the results obtained, it is possible to conclude that the nanoparticles derived from the particulate matter are magnetite (Fe_3_O_4_) nanoparticles. Even after multiple washes and muffling, the nanoparticles still presented impurities. However, when applied to microalgae harvesting, they achieve HE % = 86% (MNPs concentration = 1250 mg L^–1^; pH = 3). When functionalized with tannin, they predominantly exhibited intermolecular interactions, such as hydrogen bonding and dipole–dipole interactions. The functionalization made the particles less negatively charged, favoring their interaction with microalgae, especially at acidic pH (HE % = 77%; MNP-TANs [ ] = 1100 mg/L; pH = 3.5), reducing the number of nanoparticles. Functionalization also contributed to particle stabilization, increasing the number of reuse cycles from 3 to 7. At pH 10 (the final pH of the microalgae cultivation), both exhibited a similar HE % of 60%. These findings provide unpublished evidence that nanoparticles derived from particulate material, even without a high purity, can be efficiently applied to *Chlorella* sp. harvesting, achieving satisfactory results during various application cycles.

Beyond demonstrating satisfactory performance of the tested nanomaterials, this work also has broader implications for enabling sustainable microalgae bioprocessing. Repurposing nanoparticles sourced from iron-rich particulate matter transforms an air-quality liability into a value-added product, potentially lowering both the cost and environmental footprint relative to fully synthetic nanoparticles. Because the approach is rapid and efficient, it is a strong candidate for scale-up; however, deployment should be supported by rigorous life cycle and techno-economic assessments, as well as head-to-head comparisons with incumbent flocculants, to demonstrate viability at the industrial scale.

## Supplementary Material



## Notes

The main potential sources of magnetic iron selected in the literature; and the results of the analysis of variance (ANOVA) for the full factorial magnetic nanoparticles (MNPs) and magnetic nanoparticles functionalized with tannin (MNP-TANs) (PDF)
