# Supplementary material for: Harvesting Microalgae Biomass Using Magnetic Nanoparticles from Iron-Rich Particulate Material
Source: ACS Omega. 2026 Feb 9;11(7):11104–13. doi: 10.1021/acsomega.5c03941 (PMC12946995; doi:10.1021/acsomega.5c03941)
Supplement: Supplementary file 1 [file ao5c03941_si_001.pdf]

Supplementary material

Harvesting microalgae biomass using magnetic nanoparticles from iron-rich particulate material

*Ana Carolina de Lima Barizão[1,5], Larissa Lamburghini Brandão[2,5], Giovanna Pinto Pires[3,5]; Luiz Eduardo de Oliveira Gomes[6,7], Jairo Pinto de Oliveira[4,5], Sérvio Túlio Cassini[1,5].

[1]Federal University of Espírito Santo, Department of Environmental Engineering, Fernando Ferrari avenue, 514, Cep: 29075-910, Vitória – ES, Brazil.
[2]Federal Institute of Espírito Santo, Department of Industrial Chemistry, Min. Salgado Filho avenue, 1000, Cep: 29106-010, Vila Velha, ES, Brazil.
[3]Federal Institute of Espírito Santo, Department of Biotechnology (Renorbio-UFES), Maruípe avenue, Cep: 29053-360, Vitória – ES, Brazil.
[4]Federal University of Espírito Santo, Department of Morphology, Maruípe Avenue, Cep: 29053-360, Vitória – ES, Brazil.
[5]Research, Innovation and Development Center, Laboratory of Physical, Chemical and Microbiological Characterization, Eliezer Batista Avenue, Cep: 29140-500, Cariacica – ES, Brazil.
[6]Postgraduate Program in Environmental Oceanography (PPGOAM), Department of Oceanography and Ecology, Federal University of Espírito Santo, Cep: 29075-910, Vitória-ES, Brazil.
[7]Laboratory of Environmental Geochemistry and Marine Pollution, Department of Oceanography and Ecology, Federal University of Espírito Santo, Fernando Ferrari Avenue, 514, Cep: 29075-910, Vitória- ES, Brazil.

Corresponding author: *Ana Carolina de Lima Barizão (carolina.barizao@gmail.com)

Table S1. The main potential sources of magnetic iron selected in the literature.

Material	Iron content	Positive	Negative	Costs
Blast Furnace Slag [6]	0.45 % (FeO)	-Great availability;<br>-High information;<br>-It`s a residue.	-Low magnetic iron content;<br>-Hard processing due high carbonate content;<br>-High water consume to processing samples;	R$5.00/ tons
Blast furnace Mud [7]	41.76 % (Fe_2O_3)	-Small size (59.5% <0.038 mm);<br>-Highly disponible;<br>-High magnetic iron content;	-Presence of heavy metals;<br>-Contamination of microalgae biomass;	-Sale value not found
Sínter [21]	57.5 (Fe)	-Highly disponible;<br>-High magnetic iron content;	-It`s not a residue;<br>-Very required in steel production;	U$61.23/tons
Electrostatic precipitator dust [22]	6~10 (Fe_2O_3)	-Highly disponible;<br>-High magnetic iron content;<br>-It`s a residue.	-Can be collected and reinserted in the company itself;	-Sale value not found
Particulate material	4% (Fe_3O_4)	-Highly disponible;<br>- Don´t have costs.	-Little information available;	-Don´t have costs.

Table S2. Results of the analysis of variance (ANOVA) from the full factorial magnetic nanoparticles (MNPs). Significant p-value in **bold**.

Factor	SS	df	MS	F	p
pH (L)	**1310.296**	**1**	**1310.296**	**17.93734**	**0.002856**
pH (Q)	0.003	1	0.003	0.00004	0.994814
MNPs [ ] (L)	**439.185**	**1**	**439.185**	**6.01224**	**0.039813**
MNPs [ ] (Q)	**544.003**	**1**	**544.003**	**7.44715**	**0.025888**
Error	584.388	8	73.049		
Total SS	2971.419	12			

Table S3. Results of the analysis of variance (ANOVA) from the full factorial magnetic nanoparticles functionalized with tannin (MNP-TANs). Significant p-value in **bold**.

Factor	SS	df	MS	F	p
pH (L)	**390.4267**	**1**	**390.4267**	**21.66062**	**0.001636**
pH (Q)	63.2083	1	63.2083	3.50676	0.098009
MNP-TANs [ ] (L)	**160.8563**	**1**	**160.8563**	**8.92420**	**0.017404**
MNP-TANs [ ] (Q)	50.6589	1	50.6589	2.81052	0.132173
Error	144.1978	8	180247		
Total SS	778.2455	12			
